# An effort toward molecular biology of food deprivation induced food hoarding in gonadectomized NMRI mouse model: focus on neural oxidative status

**DOI:** 10.1186/s12868-018-0461-9

**Published:** 2018-09-24

**Authors:** Noushin Nikray, Isaac Karimi, Zahraminoosh Siavashhaghighi, Lora A. Becker, Mohammad Mehdi Mofatteh

**Affiliations:** 1 Laboratory of Molecular and Cellular Biology 1214, Department of Basic Veterinary Sciences, School of Veterinary Medicine, Razi University, Kermanshah, Iran; 2 Department of Biology, Faculty of Science, Razi University, Kermanshah, 67149-67346 Iran; 3 Department of Pathobiology, Faculty of Veterinary Medicine, Razi University, Kermanshah, Iran; 4 Department of Psychology, University of Evansville, Evansville, IN 47722 USA; 5 Department of Accounting, School of Economics and Accounting, Islamic Azad University South Tehran Branch, Tehran, Iran

**Keywords:** Gonadectomy, Hoarding behavior, Neural tissue, Oxidative stress

## Abstract

**Background:**

Environmental uncertainty, such as food deprivation, may alter internal milieu of nervous system through various mechanisms. In combination with circumstances of stress or aging, high consumption of unsaturated fatty acids and oxygen can make neural tissues sensitive to oxidative stress (OS). For adult rats, diminished level of gonadal steroid hormones accelerates OS and may result in special behavioral manifestations. This study was aimed to partially answer the question whether OS mediates trade-off between food hoarding and food intake (fat hoarding) in environmental uncertainty (e.g., fluctuations in food resource) within gonadectomized mouse model in the presence of food deprivation-induced food hoarding behavior.

**Results:**

Hoarding behavior was not uniformly expressed in all male mice that exposed to food deprivation. Extended phenotypes including hoarder and non-hoarder mice stored higher and lower amounts of food respectively as compared to that of low-hoarder mice (normal phenotype) after food deprivation. Results showed that neural oxidative status was not changed in the presence of hoarding behavior in gonadectomized mice regardless of tissue type, however, glutathione levels of brain tissues were increased in the presence of hoarding behavior. Decreased superoxide dismutase activity in brain and spinal cord tissues and increased malondialdehyde in brain tissues of gonadectomized mice were also seen.

**Conclusions:**

Although, food deprivation-induced hoarding behavior is a strategic response to food shortage in mice, it did not induce the same amount of hoarding across all colony mates. Hoarding behavior, in this case, is a response to the environmental uncertainty of food shortage, therefore is not an abnormal behavior. Hoarding behavior induced neural OS with regard to an increase in brain glutathione levels but failed to show other markers of neural OS. Decreased superoxide dismutase activity in brain and spinal cord tissues and increased malondialdehyde levels in brain tissues of gonadectomized mice could be a hallmark of debilitated antioxidative defense and more lipid peroxidation due to reduced amount of gonadal steroid hormones during aging.

## Background

Based on natural selection theory, animals in their natural habitats should adapt to varying environmental conditions for survival [1] and their adaptation may have morphological, physiological or behavioral features [2]. The major adaptive strategies of animals to environmental uncertainty include decrease of energy costs and its maintenance, migration, weight gain, hibernation or aestivation, increasing fat storage and at least hoarding behavior [2–4]. Hoarder animals prefer to either save food for the future or consume food rapidly [5]. Generally, hoarding is influenced by environment, internal milieu of animals, and their interactions. Environmental uncertainties such as food shortage, low food availability, coldness, and short day-length trigger hoarding [3, 6]. Furthermore, internal factors such as endogenous fat depots, internal energy, gonadal steroids, metabolic hormones, glucocorticoids, neuropeptide regulators of food intake, and catecholamines especially dopamine are all known to alter hoarding behavior [6–9]. However, it remains an open question whether oxidative stress (OS) mediates the trade-off between food hoarding and food intake (fat hoarding) in times of food shortage as an example of environmental uncertainty.

To the best of our knowledge, oxidative status of the central nervous system (CNS) was not broadly investigated in human hoarders or animal models. More recently, our laboratory reported an increase (~ 50-fold) in encephalic xanthine oxidoreductase (*XOR*) gene, as a key player in cellular oxidative status, in female high-hoarder *vs.* female low-hoarder while a decrease (0.026-fold) in encephalic *XOR* in male high-hoarder *vs.* male low-hoarder mice [10]. Accordingly, we concluded that food deprivation is associated with sex-dependent alteration in *XOR* expression. The OS is caused by an imbalance between production of oxidants (e.g., reactive oxygen species: ROS) and antioxidants (e.g., glutathione: GSH) and leads to progressive loss of control over biological homeostasis or rheostasis that results in functional impairments and cell death [11]. Antioxidative capacity decreases in almost all aged mammals [12, 13]. Free radicals are formed in the CNS as part of normal metabolic processes [14], while high oxygen uptake and low antioxidative defenses increase vulnerability of CNS to OS [13]. It is known that neuronal susceptibility to OS can be affected by steroid hormones. In this context, Ahlbom and coworkers demonstrated that testosterone triggers antioxidative defenses of cerebellar granule cells [15].

Food hoarding behavior has marked associations with endocrine system output, especially sex steroid hormones. For instance, Nyby and coworkers demonstrated that castration increased food hoarding in male Mongolian gerbil (*Meriones unguiculatus* Milne-Edwards, 1867) [16]. Likewise, infantile food and water deprivation resulted in impaired testicular development and lower androgen levels which may increase hoarding behavior [16]. Hence, the aim of this study was to investigate the relationship between food hoarding behavior and oxidative status of neural tissue in gonadectomized mouse model.

## Methods

### Animal housing

Male Naval Medical Research Institute (NMRI) mice (*Mus musculus* L.; *n* = 80; 2-month-old; 30–40 g) were prepared from our historical colony (Laboratory Animal House, School of Veterinary Medicine, Razi University, Kermanshah, Iran) and housed in metallic cages carpeted with wood shavings in groups consisting of 5–8 individuals. The room temperature was 22 ± 1 °C and 12 h light and dark cycles were maintained. All animals had ad libitum access to tap water and commercial standard rodent pelleted diet (Dan-e-pars Co., Iran). Animal maintenance and all research protocols were approved and reviewed by ethical committee of Razi University and followed the NIH Guide for the Care and Use of Laboratory Animals.

### Hoarding test

**Fig. 1:**
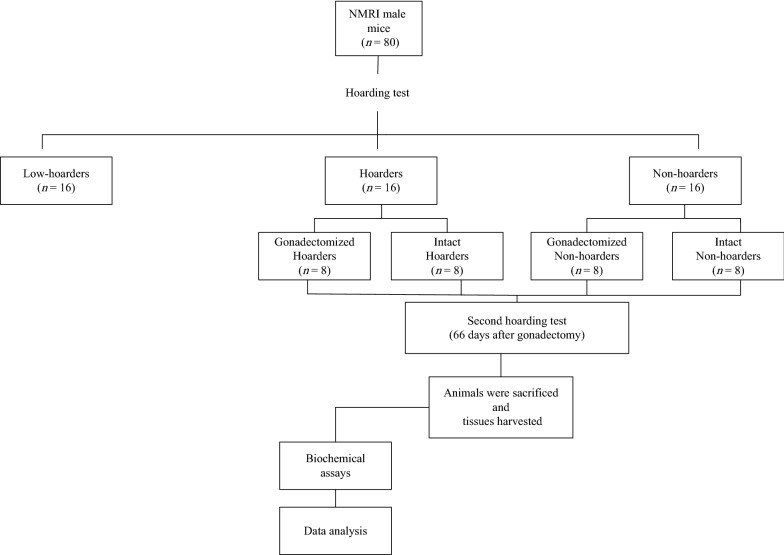
Flow chart of experimental procedure

Evaluation of hoarding behavior was performed using hoarding apparatus that was shown in Fig. 1. It composed of medium-density fibreboard (MDF) to make a home furnished with 4 chambers (13.0 × 32.0 × 6.5 cm^3^) and a hole (4 cm) in the front of each box. In addition, four wire-mesh end-sealed tubes used as food storing tubes (45 cm long, 4 cm external diameter). The wire-meshes were rolled in order to prevent food pellet dropping. Plastic tubes (10 cm long) were embedded in holes of boxes and storing tubes connected to them. The proximal end of hole was blocked with a removable plastic plug. A wire mesh used as the roof of MDF made home. Each box was equipped with a water bottle. For at least one night, we considered a limestone-made lodge in home box which furnished by wood shaving bedding to simulate natural nest for mice (Fig. 1).

Each mouse weighed and introduced to home box in the morning. Food pellets (100 g) were placed in the wire-mesh tube (storing tube) while access to food was restricted via an interface plastic plug. The plugs were removed just before the start of the dark and food-restricted mice were allowed to access obtainable food in storing tube. The next morning, body weights and weight of food pellets which each mouse had been hoarded into the home base box were recorded. Of course animals had ad libitum access to water during the experiment [17]. Mice that hoarded less than 5 g of food were considered “non-hoarders” while mice that hoarded more than 20 g of food were considered “hoarders” [10]. Mice that hoarded between 5 and 20 g of food were considered as “low-hoarder” subjects and excluded from study.

### Gonadectomy

After screening of hoarder (*n *= 16) and non-hoarder (*n *= 16) mice using hoarding apparatus (vide supra), half of the mice were randomly underwent gonadectomy. The model used in present study was accelerated aging and animals were gonadectomized through bilateral orchiectomy. For this purpose, animals were anesthetized by intraperitoneal (i.p.) injection of ketamine (80 mg/kg)/diazepam (0.5 mg/kg) cocktail. During surgical procedure, animals receiving abdominal incision and both testes and their associated epididymides were removed.

### Treatment groups

**Fig. 2:**
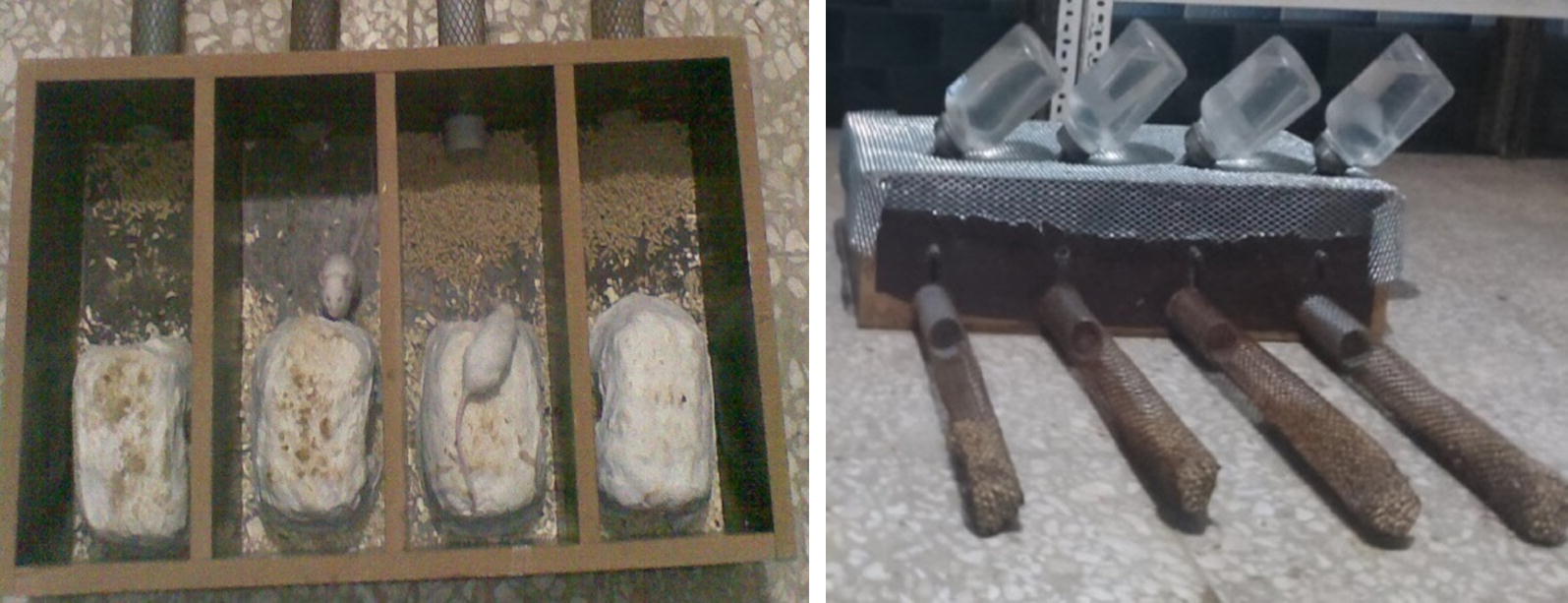
Manufactured apparatus used to screen hoarding behavior. Right photo shows empty apparatus while left photo shows mice while hoarding

Mice were divided into four groups of eight including intact hoarder, gonadectomized hoarder, intact non-hoarder and gonadectomized non-hoarder male mice. For comparison, hoarding behavior between gonadectomized and intact mice, hoarding test was repeated 66 days post-gonadectomy (Fig. 2). Biochemical assays were performed only on hoarder and non-hoarder mice (vide infra).

### Preparation of tissue homogenates

All mice in our screened population were weighed and sacrificed by decapitation using a guillotine without anesthesia after 12–14 h fasting. Brain and spinal cord tissues have been harvested over ice blocks, weighed, wrapped in aluminum foil, snap-frozen in liquid nitrogen and stored at − 70 °C until use for biochemical assays.

Frozen brains and spinal cords were thawed and homogenized over ice by a tissue homogenizer (WiseTis model HG-15D; Korea) in potassium phosphate buffer solution (50 mM; pH 7.4) for 3 times, at 3,000 rpm. Then, tissue homogenates were centrifuged at 12,000 *g* at 4 °C for 15 min using super-speed refrigerated centrifuge bridges (Sigma, model 3–30 K; Germany) and resulting tissue extracts (supernatants) were stored at − 20 °C for biochemical assays [18].

### Estimation of glutathione (GSH) levels

Trichloroacetic acid (TCA; 5%; 0.5 ml) solution was added to tissue extract (0.5 ml) to precipitate proteins and centrifuged at 3000 rpm for 20 min. Sodium phosphate buffer solution (1 ml; pH 8.0) and 5,5’-dithiobis(2-nitrobenzoic acid) (DTNB; 1 mM; 0.5 ml) were added to the supernatant (0.1 ml). The absorbance of the yellow color developed was measured at 412 nm [19]. The GSH concentration (µM) was determined using GSH dissolved in diluted metaphosphoric acid as standard.

### Estimation of catalase (CAT) activity

The CAT (hydrogen peroxide oxidoreductase) activity was assayed based on its ability to oxidize hydrogen peroxide [20]. Briefly, potassium phosphate buffer (2.25 ml; 65 mM; pH 7.8) was mixed to tissue extract (0.1 ml) and incubated at 25 °C for 30 min. Then, 3 ml of hydrogen peroxide (30% w/v) was added to the mixture and the decline in absorbance was measured at 240 nm for 3 min by UV–Vis spectrophotometer [21]. The CAT activity was measured based on its ability to decompose 1 mM of hydrogen peroxide per min per protein (mg) at 25 °C.

### Estimation of superoxide dismutase (SOD) activity

The activity of superoxide dismutase was determined based on methodology of Misra and Fridowich (1977) with some modifications [22]. Nitroblue tetrazolium (NBT; 0.4 ml; 25 mM), and hydroxylamine HCl (0.02 ml; 0.1 mM) were added to sodium bicarbonate solution (1 ml; 50 mM). Tissue extract (0.1 ml) was added to the mixture and its absorbance was measured at 560 nm after 2 min using UV–Vis spectrophotometer. The SOD activity was measured based on its ability to prevent photo-reduction of 1 mM of NBT per min per protein (mg) at 25 °C.

### Estimation of total antioxidant status (TAS)

Concisely, 0.1 ml of tissue extract was deproteinated using 1 ml of methanol, vortexed for 30 s, then centrifuged at 3000 rpm for 30 min. Stable free radical *α,α*-diphenyl-*β*-picryl hydrazyl (DPPH; 0.5 ml; 0.2 mM) was prepared in methanol (1.5 ml), then added to the supernatant, mixed thoroughly and absorbance was measured at 517 nm against blank. The standard graph was plotted using different concentrations of ascorbic acid and the antioxidative status values were expressed in terms of nM of ascorbic acid [23].

### Estimation of lipid peroxidation (malondialdehyde (MDA) assay)

The magnitude of lipid peroxidation was determined by measuring MDA which is thiobarbituric acid reactive substance (TBARS). To precipitate the proteins, TCA (30%; 0.5 ml) was added to tissue extract (0.5 ml), vortexed for 30 s, and finally centrifuged at 3000 rpm for 5 min. Thiobarbitoric acid (TBA; 1%; 500 µl) solution and 500 µl of distilled water were added to the supernatant and the resulting mixture heated for 1 h at 98 °C, then cooled to room temperature and kept in ice for 5 min. At last, the absorbance of pink mixture recorded at 532 nm. Standard graph was plotted using 1,1,3,3-tetraethoxy propane (TEP) to estimate MDA values [24].

### Protein content of tissue extract

Each assay was performed at least in triplicate and protein concentration was determined by the method of Bradford (1976) using bovine serum albumin as the standard [25].

### Statistical analyses

Statistical analyses performed using SPSS version 20.0 software for Windows (SPSS, Chicago, IL, USA). Normal distribution of data was assessed using the Shapiro–Wilk normality test. The data with normal distribution were analyzed using one- or two-way analysis of variance (ANOVA) and data were not normally distributed, submitted to nonparametric statistics, Kruskal–Wallis H test. Post hoc LSD test was used for additional comparison. Body weights were compared before and after hoarding evaluation, using paired samples *T* test. Pearson correlation test was used for evaluation of the correlation (r) between the body weight before and after the hoarding with the amount of hoarded food. A *p* value ≤ 0.05 was considered as statistically significant and results were expressed as mean ± standard error of the mean (SEM).

## Results

### Hoarding test

Classifying of animals into hoarder, low-hoarder and non-hoarder groups showed that there was no significant correlation between the body weight before hoarding and the amount of hoarded food in screened population (r = 0.274, *p* = 0.097). There was also no significant relationship between body weight after hoarding and the amount of hoarded food (r = 0.056, *p* = 0.737). However, hoarder, low-hoarder and non-hoarder groups had significant difference based on amounts of hoarded food (F(2,37) = 47.520, *p* = 1.057e^−10^). Post hoc LSD test showed that there was a significant difference in amount of hoarded food between hoarder group in comparison with low hoarder group (*p* = 3.475e^−6^) and non-hoarder group (*p* = 2.144e^−11^; Table 1). Also the low-hoarder group compared to non-hoarder group (*p* = 0.016) had significant increase in amount of hoarded food (Table 1**)**.

**Table 1:** Hoarded food (g) in food deprived colony mate mice used to classify mice based on hoarding test

Parameter	P_ANOVA_	F(2,37)	Groups		
			Hoarder	Low-hoarder	Non-hoarder
BW (g) before hoarding	0.378	1.000	23.92 ± 0.95	21.62 ± 1.28	22.81 ± 0.97
BW (g) after hoarding	0.572	0.567	22.64 ± 1.29	22.12 ± 1.74	23.93 ± 0.89
Hoarded food (g) in day 0	1.057e−10	47.520	37.35 ± 4.41a	12.37 ± 1.32b	1.20 ± 0.32c

Results shown as mean ± SEM and parameters that are significantly different at P_ANOVA_ ≤ 0.05 are displayed with different letters among groups

*BW* body weight

This significant difference in the amounts of hoarded food among these groups confirmed precise classification based on hoarded food in colony. There was no significant difference between the animal’s body weight before and after hoarding using paired-samples T-test (t = −  0.234, *p* = 0.817, df = 37) which indicated food intake during the hoarding test was not different among groups. There was no significant difference in the amount of hoarded food between gonadectomized and intact mice in second hoarding test at the end of study (F(1,10) = 0.534, *p* = 0.483). However, the amount of hoarded food by gonadectomized mice was tended to be higher than intact mice (data not shown).

### The interaction between gonadectomy and the presence of hoarding behavior on neural antioxidative profile

Using two-way ANOVA, it has been revealed that tissue, gonadectomy, presence or absence of hoarding behavior and the interaction between them have not a significant impacts on the CAT, SOD, TAS, MDA, and total protein content in the neural tissue while GSH levels were significantly varied in tissues (F(1,29) = 51.683, *p* = 0.039; Table 2).

**Table 2:** Interaction between gonadectomy and hoarding behavior on glutathione levels (µM) in neural tissues

Parameter	F(1,29)	P_ANOVA_
Glutathione	2.998	0.333
Hoarding	1.697	0.417
Gonadectomy	0.895	0.518
Tissue	51.683	0.039*
Hoarding × gonadectomy	0.582	0.585
Hoarding × tissue	3.891	0.299
Gonadectomy × tissue	7.409	0.224
Hoarding × gonadectomy × tissue	0.137	0.714

Parameters with significant difference at P_ANOVA_ ≤ 0.05 in each row have marked with * sign

### Evaluation of neural antioxidant status

The GSH levels had significant difference in brain tissues of studied groups (F(3,17) = 3.926, *p* = 0.032; Table 3). Post hoc LSD test showed that GSH levels were significantly higher in brain tissues of intact hoarder group in comparison with intact non-hoarder group (*p* = 0.007) and in gonadectomized hoarder group in comparison with intact non-hoarder group (*p* = 0.022).

**Table 3:** Glutathione levels in the brain tissues of studied groups

Group	Glutathione (µM)
Intact hoarder	1428.78 ± 22.17ac
Intact non-hoarder	1292.99 ± 22.96b
Gonadectomized hoarder	1414.01 ± 44.51c
Gonadectomized non-hoarder	1355.41 ± 23.69ab

Results shown as mean ± SEM; in columns, values with different letters are significantly different

GSH levels of spinal cord tissues showed no significant differences among groups (F(3,18) = 0.509, *p* = 0.682). The CAT levels in the brain tissues (F(3,13) = 1.062, *p* = 0.408) and spinal cord (F(3,13) = 1.582, *p* = 0.255) were not different in studied groups. The SOD levels did not show any significant difference in the brain tissues of the studied groups (F(3,19) = 1.960, *p* = 0.161). Post hoc LSD test showed a significant reduction in the SOD levels of brain in gonadectomized non-hoarder group compared to intact non-hoarder group (*p* = 0.048). No significant difference was found in the SOD levels of spinal cord among studied groups (F(3,20) = 2.053, *p* = 0.145). However, a significant reduction in the SOD levels of spinal cord in gonadectomized non-hoarder group compared with intact non-hoarder group was observed using Post hoc LSD test (*p* = 0.049). There was no significant differences in TAS of brain (F(3,18) = 1.158, *p* = 0.358) and spinal cord (F(3,20) = 1.004, *p* = 0.415) tissues of studied groups. The amount of MDA in the brain tissues showed no significant difference between groups (F(3,19) = 2.818, *p* = 0.072). Post hoc LSD test showed that MDA levels in the brain of gonadectomized non-hoarder group has been significantly increased in comparison with intact hoarder group (*p* = 0.034). The MDA levels in the spinal cord had no significant difference between groups (F(3,23) = 0.872, *p* = 0.472). The protein content of brain tissues did not show any significant change in studied groups (F(3,21) = 2.022, *p* = 0.147). Shapiro–Wilk normality test showed that protein content in spinal cord tissues were not normally distributed, so the non-parametric Kruskal–Wallis H test was used. Total protein content in the spinal cord of studied groups showed no significant difference (Chi square = 1.749, asymptotic significance = 0.626, df = 1).

### Effect of hoarding on neural antioxidative profile

Presence of hoarding behavior had significant effect on the GSH levels in the brain tissues of studied mice (F(1,17) = 9.66, *p* = 0.007). This means that the GSH levels in the brain tissues of hoarder mice were higher than those of non-hoarder mice (Table 4).

**Table 4:** Effect of hoarding on antioxidative profile of brain tissues in male mice

Parameter	Group	Quantity	F value	P_ANOVA_
GSH	Hoarder Non-hoarder	1422.87 ± 20.83 1332.00 ± 19.68	F(1,17) = 9.66	0.007*
CAT	Hoarder Non-hoarder	0.148 ± 0.067 0.219 ± 0.057	F(1,13) = 0.639	0.440
SOD	Hoarder Non-hoarder	4.04 ± 0.411 3.39 ± 0.512	F(1,19) = 0.979	0.336
TAS	Hoarder Non-hoarder	1229.36 ± 40.86 1226.75 ± 38.49	F(1,18) = 0.002	0.965
MDA	Hoarder Non-hoarder	47.76 ± 4.16 51.07 ± 4.49	F(1,19) = 0.276	0.606
Protein	Hoarder Non-hoarder	2.38 ± 0.046 2.21 ± 0.071	F(1,21) = 4.327	0.056

Data are expressed as mean ± SEM and parameters with P_ANOVA_ ≤ 0.05 are significantly different and displayed with * sign

*GSH* glutathione (µM), *CAT* catalase (1 mM of H2O2/min/mg protein), *SOD* superoxide dismutase (1 mM of NBT/min/mg protein), *TAS* total antioxidant status (nM of ascorbic acid), *MDA* malondialdehyde (nM/mg protein), protein (mg/ml)

Hoarding behavior had no significant effect on the antioxidative profile of spinal cord tissue (Table 5). Shapiro–Wilk normality test showed that protein content in spinal cord tissues was not normally distributed, therefore the protein content in the spinal cord of male mice were analyzed using Kruskal–Wallis H test. Hoarding behavior had no significant effect on the protein content of spinal cord tissue; hoarder mice (1.901 ± 0.097) vs non-hoarder mice (1.857 ± 0.091; Chi Square = 0.431; Asymp. Sig = 0.511; df = 1).

**Table 5:** Effect of hoarding on antioxidative profile of spinal cord tissues male mice

Parameter	Group	Quantity	F value	P_ANOVA_
GSH	Hoarder Non-hoarder	5447.98 ± 558.67 4838.94 ± 482.72	F(1,18) = 0.547	0.470
CAT	Hoarder Non-hoarder	1.62 ± 0.380 1.057 ± 0.203	F(1,13) = 1.957	0.187
SOD	Hoarder Non-hoarder	22.12 ± 3.42 19.76 ± 3.85	F(1,20) = 0.209	0.653
TAS	Hoarder Non-hoarder	9078.00 ± 1095.09 9859.66 ± 1937.34	F(1,20) = 0.139	0.713
MDA	Hoarder Non-hoarder	441.26 ± 69.09 389.59 ± 72.00	F(1,23) = 0.266	0.611

Data are expressed as mean ± SEM and parameters with P_ANOVA_ ≤ 0.05 are significantly different and displayed with * sign

*GSH* glutathione (µM), *CAT* catalase (1 mM of H2O2/min/mg protein), *SOD* superoxide dismutase (1 mM of NBT/min/mg protein), *TAS* total antioxidant status (nM of ascorbic acid), *MDA* malondialdehyde (nM/mg protein)

### Effect of gonadectomy on neural antioxidative profile of male mice

Amongst all studied parameters of antioxidative profile, MDA levels in brain tissues were significantly affected by gonadectomy (F(1,19) = 9.067, *p* = 0.008). MDA levels were higher in brain tissues of gonadectomized mice than intact mice (Table 6).

**Table 6:** Effect of gonadectomy on antioxidative profile of brain tissues in male mice

Parameter	Group	Mean ± SEM	F value	P_ANOVA_
GSH	Gonadectomized	1383.51 ± 27.58	F(1,17) = 0.003	0.956
	Intact	1381.45 ± 24.33		
CAT	Gonadectomized	0.240 ± 0.068	F(1,13) = 2.501	0.140
	Intact	0.108 ± 0.027		
SOD	Gonadectomized	3.48 ± 0.398	F(1,19) = 1.118	0.304
	Intact	4.16 ± 0.517		
TAS	Gonadectomized	1275.00 ± 32.51	F(1,18) = 3.55	0.077
	Intact	1176.33 ± 41.73		
MDA	Gonadectomized	55.99 ± 3.38	F(1,19) = 9.067	0.008*
	Intact	40.65 ± 3.82		
Protein	Gonadectomized	2.36 ± 0.063	F(1,21) = 1.512	0.233
	Intact	2.26 ± 0.055		

Data are expressed as mean ± SEM and parameters with P_ANOVA_ ≤ 0.05 are significantly different and displayed with * sign

*GSH* glutathione (µM), *CAT* catalase (1 mM of H2O2/min/mg protein), *SOD* superoxide dismutase (1 mM of NBT/min/mg protein), *TAS* total antioxidant status (nM of ascorbic acid), *MDA* malondialdehyde (nM/mg protein), protein (mg/ml)

Gonadectomy had significant effect on the SOD levels in spinal cord tissue (F(1,20) = 5.45, *p* = 0.031) but not on any other measure. In this regard, SOD levels in gonadectomized mice have been significantly decreased in comparison with intact mice (Table 7**)**.

**Table 7:** Effect of gonadectomy on antioxidative profile of spinal cord tissues in male mice

Parameter	Group	Mean ± SEM	F value	P_ANOVA_
GSH	Gonadectomized	4900.63 ± 644.09	F(1,18) = 0.742	0.401
	Intact	5582.44 ± 426.33		
CAT	Gonadectomized	1.37 ± 0.349	F(1,13) = 0.179	0.679
	Intact	1.19 ± 0.161		
SOD	Gonadectomized	16.52 ± 2.98	F(1, 20) = 5.45	0.031*
	Intact	27.23 ± 3.49		
TAS	Gonadectomized	8386.33 ± 1583.51	F(1,20) = 1.39	0.252
	Intact	10781.88 ± 994.22		
MDA	Gonadectomized	357.16 ± 64.59	F(1,23) = 2.23	0.149
	Intact	502.16 ± 70.63		

Data are expressed as mean ± SEM and parameters with P_ANOVA_ ≤ 0.05 are significantly different and displayed with * sign

*GSH* glutathione (µM), *CAT* catalase (1 mM of H2O2/min/mg protein), *SOD* superoxide dismutase (1 mM of NBT/min/mg protein), *TAS* total antioxidant status (nM of ascorbic acid), *MDA* malondialdehyde (nM/mg protein)

Gonadectomy had no significant effect on the overall protein content in spinal cord tissue. Shapiro–Wilk normality test showed that protein content in spinal cord tissues was non-parametrically distributed, so analysis was carried out with Kruskal–Wallis H test; gonadectomized mice (1.813 ± 0.080) and intact mice (1.995 ± 0.104) did not differ (Chi Square = 1.269; Asymp. Sig = 0.260; df = 1).

## Discussion

Hoarding behavior is more prevalent in older adults [26]. The CNS is exclusively vulnerable to oxidative damage because of its high energy needs, oxygen consumption, and iron content and relatively low efficient antioxidative systems [27]. In addition, the ability of brain to repair its damaged cells is curtailed with age because it composed of postmitotic neurons and differentiated glial cells [28]. If the amount of free radicals in brain was in their physiological levels, the innate antioxidative defense neutralizes this assault. Otherwise, CNS antioxidative defense against oxidants fails and subsequently, the activity of neurons as well as cognitive functions will be severely affected [29]. Many attempts have been made to translate hoarding behavior in animal models, however a thorough and reliable mechanism and model has not been identified. In this study, examined a potential relationship between hoarding behavior and oxidative status in a gonadectomized mouse model.

We did not find any significant difference between body weights of mice before and after hoarding screening. Since weight control is a long-term physiological phenomenon so within 12 h of fasting and access to food did not change. Studied groups had a significant difference in amount of hoarded food in day 0. This difference in hoarding behavior among colony members remains unanswered by us and other previous studies [5, 6, 10, 17, 30–32]. Previous studies have focused more on presenting and describing hoarding behavior while this study initially has a molecular insight to antioxidative status in the presence of hoarding behavior. This study suggests that changes in antioxidative status between individuals may be the cause of these differences. Since behavioral changes are consequence of overt biological changes, we excluded the low-hoarder group from the study so that we decide to find biological connection in both lower and upper boundaries of hoarding expression.

Animals have an innate defense system including endogenous antioxidants to neutralize harmful effects of oxidative insults [33]. It is noteworthy that the glutathione is one of the most important antioxidants found in cells [34]. This tripeptide (Glu-Cys-Gly) contains cysteine residue which its “thiol” group acts as a reducing agent [35] and converts free radicals into non-toxic substances such as water and oxygen [36]. Glutathione exists in 2 forms: reduced (GSH) and oxidized (GSSG), which an increase of GSSG to GSH is an index of OS [37]. Disturbance in glutathione metabolism complicates the pathogenicity of various psychiatric disorders [38–40]. It seems that GSH levels in neural tissues has tissue-specific activity which may be due to difference in protein contents of neural tissues. In present study, GSH levels have been significantly increased in brain tissues of intact and gonadectomized hoarders in comparison to intact non-hoarders. One of striking feature of present study was higher GSH levels in brain tissues of hoarders in comparison with non-hoarders. This finding may be due to the functional changes of gamma-glutamylcysteine synthetase and GSH synthetase or through the interference in de novo GSH synthesis by glutathione reductase function [41]. If hoarding is considered a “coping behavior” which occurs in response to stresses, such as food shortage [42], then increased antioxidant levels could be a mechanism used to confront this stress.

The brain consumes about 20% of total body oxygen [43] which increases the possibility of superoxide production. The SOD has a key role in antioxidative defense and mice lacking SOD enzyme die a few days after birth [44]. Three main families of SOD are known in human and most vertebrates which are categorized based on their relevant metal cofactor and cellular location [45, 46]. In this line, SOD1 (Cu/Zn SOD) is found in cytoplasm, SOD2 (Mn SOD) is located in mitochondria, and SOD3 (Cu/Zn SOD) is active in extracellular space [47]. In present study, SOD levels were significantly lower in brain and spinal cord tissues of gonadectomized non-hoarders than intact non-hoarders. The SOD activity was significantly lower in spinal cord tissues of gonadectomized mice in comparison with intact mice. Accordingly, we can conclude that induction of gonadectomy in mice is one of the main factors that reduces SOD activity in neural tissues of gonadectomized mice. Androgens have both neuroprotective [47–49] and neurotoxic effects [50] depending on biological systems and their concentrations [46, 48]. For instance, testosterone has antioxidative properties in human prostate [51] and rat nervous system [15, 52] nevertheless high testosterone levels produce oxidation in testicular tissues of rats and rabbits [53, 54], muscles of rats [55], and placenta of women [56]. These findings indicate that pro-oxidative effect of testosterone depends on type of tissue and testosterone levels [57]. Neuroprotective properties of testosterone may be related to its conversion to estradiol that has protective role on dopamine neurons in experimental studies [58–60]. It seems that either directly or through aromatization to estrogen, testosterone exerts its protective effect on nerve cells [61]. Gonadectomy is associated with reduction of testosterone, dihydrotestosterone and estradiol levels. These hormones have a key role in activation of cytoplasmic SOD (SOD1) [62] and reduced levels of aforementioned hormones can be considered as main reason of reduced activity of SOD enzyme in brain and spinal cord tissues of gonadectomized mice in comparison to intact mice. In a similar study that examined the impact of orchiectomy on SOD levels in hippocampus of rat, decrease in SOD activity was detected in gonadectomized group compared to sham group that caused oxidative damage and morphological changes in hippocampal tissue [63]. In a comparative study on 21–92 years old men, an age-related decrease in SOD activity in thoracic segment and cervical intumescence of spinal cord was seen [64]. In other study, a decline in the activities of antioxidant enzymes and a non-significant decrease of SOD activity were observed in brain tissues of 4–24 months old rats [65]. However, an increase in SOD activity in various parts of neural tissue by aging has been reported in other studies [12, 66]. Taken in sum, we can conclude that SOD activity is different in various parts of CNS and decreased steroid sex hormones following gonadectomy can reduce SOD activity and subsequently lead to weakness in antioxidative defense of neural tissue.

As mentioned earlier, CAT is an enzyme that converts hydrogen peroxide to water and oxygen and this enzyme has been characterized in rat brain as a peroxisomal marker enzyme which is involved in antioxidative defense [67]. However, its main role is still unknown because mice lacking CAT seem normal [68]. In present study, CAT activity in neural tissue was varied independent to tissue, gonadectomy, hoarding behavior and their interactions. The CAT activities tended to be higher in brain and spinal cord tissues of gonadectomized mice in comparison with intact mice. In this line, an age-related increase in CAT activity in cervical and lumbosacral intumescence of spinal cord has been reported in 21–92 years old men [64]. Nonetheless, reduction in CAT activity due to aging has been reported in several studies [12, 65, 69]. The CAT activity and its mRNA in brain tissues of male Fisher rats were correlated and significantly decreased with aging [70]. Hence, CAT activity could be attributed to the expression and distribution of this enzyme in different tissues. Although the amounts of produced hydrogen peroxide in various tissues can influence CAT activity.

The MDA is an organic compound produced through decomposition of multiple unsaturated lipids and usually considered as a biomarker of lipid peroxidation [71]. The MDA reacts with free amino groups of proteins, phospholipids and nucleic acids and leads to structural changes [72]. Neuronal membranes are rich in polyunsaturated fatty acids, substrates of ROS, which increase the susceptibility of neurons to OS [73]. In present investigation, MDA levels were significantly higher in brain tissues of gonadectomized non-hoarder group than the intact hoarder group. Gonadectomy also caused a significant increase in MDA levels in brain tissues of gonadectomized mice in comparison with intact mice. In this context, chronic stresses built up lipid peroxidation and MDA production in brain tissues of rats and weakened antioxidative defense [74]. As well as estrogen and testosterone which possess antioxidative properties may be declined post-gonadectomy which culminates to membrane lipid peroxidation and MDA production [75]. In other comparable study, an increase of lipid peroxidation and MDA in hippocampus of gonadectomized rats has been reported compared to sham group [63]. Accordingly, we can conclude that probable decline in steroid sex hormones that occurred following the gonadectomy may lead to weakened antioxidative defense and increased lipid peroxidation and MDA production.

## Conclusion

Overall, our results showed that food deprivation-induced hoarding behavior in mice cannot be simply a strategic response to food shortage because all colony mates exposed to food deprivation in this study did not show hoarding behavior. On the other hand, hoarding behavior is considered a naturally occurring action in response to an environmental cue, food shortage, and not a disorder. In addition, decreased gonadal steroid hormones, which physiologically occurs during andropause, may increase the incidence of this behavior because of the gonadectomized mice hoarded non-significantly more food in comparison to intact mice. Decreased SOD activity in brain and spinal cord tissues and increased MDA levels in brain tissues of gonadectomized mice could be a sign of weakened antioxidative defense and more lipid peroxidation due to reduced amount of gonadal steroid hormones during aging. Current results showed that hoarding behavior does not have a significant impact on the neural antioxidative profile except the increase in brain GSH levels. Increased brain GSH levels in the presence of hoarding behavior may indicate the effect of this behavior on improving brain antioxidative defense or may imply on the increase of OS in the presence of this behavior. Therefore, GSH could be a good candidate in studying molecular biology of hoarding behavior.

## Abbreviations

OS: oxidative stress
CNS: central nervous system
ROS: reactive oxygen species
GSH: glutathione
NMRI: Naval Medical Research Institute
i.p.: intraperitoneal
MDF: medium-density fibreboard
TCA: trichloroacetic acid
DTNB: 5,5’-dithiobis(2-nitrobenzoic acid)
CAT: catalase
SOD: superoxide dismutase
NBT: nitroblue tetrazolium
TAS: total antioxidant status
DPPH: *α,α*-diphenyl-*β*-picryl hydrazyl
MDA: malondialdehyde
TBARS: thiobarbituric acid reactive substance
TBA: thiobarbitoric acid
TEP: 1,1,3,3-tetraethoxy propane
